# Pemetrexed/Carboplatin/Bevacizumab followed by Maintenance Pemetrexed/Bevacizumab in Hispanic Patients with Non-Squamous Non-Small Cell Lung Cancer: Outcomes according to Thymidylate Synthase Expression

**DOI:** 10.1371/journal.pone.0154293

**Published:** 2016-05-18

**Authors:** Andrés Felipe Cardona, Leonardo Rojas, Beatriz Wills, Oscar Arrieta, Hernán Carranza, Carlos Vargas, Jorge Otero, Mauricio Cuello, Luis Corrales, Claudio Martín, Carlos Ortiz, Sandra Franco, Rafael Rosell, CLICaP

**Affiliations:** 1 Clinical and Translational Oncology Group, Thoracic Oncology Unit, Institute of Oncology, Clínica del Country, Bogotá, Colombia; 2 Foundation for Clinical and Applied Cancer Research (FICMAC), Bogotá, Colombia; 3 Clinical Oncology Department, Centro Javeriano de Oncología, Hospital Universitario San Ignacio, Bogotá, Colombia; 4 Thoracic Oncology Unit, Instituto Nacional de Cancerología (INCan), México City, México; 5 Clinical Oncology Department, Hospital de Clínicas–UdeLAR, Montevideo, Uruguay; 6 Clinical Oncology Department, Hospital San Juan de Dios, San José, Costa Rica; 7 Thoracic Oncology Unit, Alexander Fleming Institute, Buenos Aires, Argentina; 8 Medical Oncology Department, Catalan Institute of Oncology, Hospital Germans Trias i Pujol, Badalona, Barcelona, Spain; University of North Carolina School of Medicine, UNITED STATES

## Abstract

**Objective:**

To evaluate the efficacy and safety of pemetrexed, carboplatin and bevacizumab (PCB) followed by maintenance therapy with pemetrexed and bevacizumab (PB) in chemotherapy-naïve patients with stage IV non-squamous non-small cell lung cancer (NSCLC) through the influence of thymidylate synthase (TS) protein and mRNA expression on several outcomes. The primary endpoints were the overall response rate (ORR), progression-free survival (PFS) and overall survival (OS).

**Methods:**

A cohort of 144 patients were administered pemetrexed (500 mg/m^2^), carboplatin (AUC, 5.0 mg/ml/min) and bevacizumab (7.5 mg/kg) intravenously every three weeks for up to four cycles. Maintenance PB was administered until disease progression or unacceptable toxicity.

**Results:**

One hundred forty-four Colombian patients with a median follow-up of 13.8 months and a median number of 6 maintenance cycles (range, 1–32) were assessed. The ORR among the patients was 66% (95% CI, 47% to 79%). The median PFS and (OS) rates were 7.9 months (95% CI, 5.9–10.0 months) and 21.4 months (95% CI, 18.3 to 24.4 months), respectively. We documented grade 3/4 hematologic toxicities, including anemia (14%), neutropenia (8%), and thrombocytopenia (16%). The identified grade 3/4 non-hematologic toxicities were proteinuria (2%), venous thrombosis (4%), fatigue (11%), infection (6%), nephrotoxicity (2%), and sensory neuropathy (4%). No grade >3 hemorrhagic events or hypertension cases were reported. OS was significantly higher in patients with the lowest TS mRNA levels [median, 29.6 months (95% CI, 26.2–32.9)] compared with those in patients with higher levels [median, 9.3 months (95% CI, 6.6–12.0); p = 0.0001]. TS expression (mRNA levels or protein expression) did not influence the treatment response.

**Conclusion:**

Overall, PCB followed by maintenance pemetrexed and bevacizumab was effective and tolerable in Hispanic patients with non-squamous NSCLC. This regimen was associated with acceptable toxicity and prolonged OS, particularly in patients with low TS expression. We found a role for Ki_67_ and TS expression as prognostic factors.

## Introduction

Lung cancer (LC) is the leading cause of cancer-related mortality worldwide. According to the International Agency for Research on Cancer (IARC), the incidence of LC in 2012 was 23.1 cases per 10^5^, while the case fatality ratio was 19.7 deaths per 10^5^. LC corresponds to 13% of all cancer cases and 19% of all cancer deaths [1]. In Colombia, LC is the third most common cancer after prostate and gastric cancer [1]. Despite the increasing efforts in novel screening techniques and global awareness, most patients are diagnosed at advanced non-surgical stages; thus, only 16.8% of patients survive at 5 years [2].

Research has highlighted the importance of LC histology on the treatment decision. The most frequent histological subtype of non-small cell lung cancer (NSCLC) is adenocarcinoma, comprising 55% of all cases [3]. The management of advanced NSCLC, particularly the non-squamous subtype, has changed in the past decade due to the discovery of molecular markers that influence the biological behavior of the disease and are now targets of cancer therapy [4,5]. For example, the identification of the activating mutation of Epidermal Growth Factor Receptor (EGFR) as a predictive feature for tyrosine kinase inhibitors (TKIs) led to the establishment of TKIs as the first-line treatment for EGFR-positive NSCLC [6–11].Additionally, the description of anaplastic lymphoma kinase (ALK) rearrangement supported the development of TKIs against ALK, which are currently the first-line therapy for patients with ALK translocation [12]. Interestingly, ALK treatments were developed faster (4 years) than the other targeted therapies for NSCLC that became available for clinical use only after 10 years [13,14].

Although molecular biology and the development of targeted therapies have significantly progressed, approximately half of the patients lack specific molecular mutations. Furthermore, even when we identify certain molecular mechanisms, specific therapies for certain common mutations are still missing (e.g., RAS) [13].

Platinum-based chemotherapy remains the standard therapy for patients lacking EGFR mutations or ALK translocations; such regimen has been shown to improve both quality of life and survival risk death reduction; HR, 0.77; (95% CI, 0.71–0.83; P = .0001) [15]. This chemotherapy in combination (doublets) is recommended for patients with NSCLC and Eastern Cooperative Oncology Group (ECOG) performance status (PS) of either 0 or 1 [16,17]. However, for a subgroup of patients with non-squamous NSCLC, certain platinum combinations are thought to be more effective than others. Platinum combinations with pemetrexed were documented to be better than other platinum-based regimens for advanced lung adenocarcinoma [18]; as such, these are the standards of care for non-squamous NSCLC [16,17]. Moreover, in different phase III trials the addition of bevacizumab to chemotherapy and its continuance as maintenance therapy improved progression-free survival (PFS) compared with chemotherapy alone [19–21]. Furthermore, patients without disease progression who received four to six chemotherapy cycles of these agents and then continued with pemetrexed with or without bevacizumab (maintenance therapy) had better outcomes as demonstrated by prolonged Progression-free survival (PFS) and increased overall survival (OS) compared with a watch and wait strategy [22–25].

There is growing interest in developing biomarkers for predictive purposes regarding LC chemotherapy. Research has suggested that thymidylate synthase (TS) expression, an enzymatic complex that participates in folate synthesis, could be used as a surrogate for the treatment response to pemetrexed [26,27]. In vitro studies have supported this theory since LC cell lines with low TS expression are highly sensitive to pemetrexed [28–30].

The main objective of this retrospective cohort was to describe the efficacy and safety of the carboplatin/pemetrexed/bevacizumab regimen (PCB) followed by maintenance therapy (in stable patients) with pemetrexed/bevacizumab (PB) in a real-life scenario of Hispanic adult patients with advanced non-squamous NSCLC. Our secondary objective was to analyze TS protein and mRNA expression and their association with clinical outcomes.

## Subjects and Methods

### Patient characteristics and tumor samples

One hundred forty-four lung adenocarcinoma chemotherapy-naïve patients who received at least one cycle of PCB (the treatment included 4 cycles of induction with pemetrexed 500 mg/m^2^, carboplatin AUC of 5, and bevacizumab 7.5 mg/kg intravenously every 3 weeks followed by maintenance pemetrexed and bevacizumab at identical doses until disease progression or intolerance) were included for the evaluation of: i) the treatment response, ii) safety, and iii) toxicity. Eligibility for the regimen was determined by the treating oncologist who considered standard bevacizumab inclusion criteria (absence of hemoptysis, hemorrhagic brain metastasis, cavitation or involvement of large blood vessels). All of the study cases had complete clinical records and available tissue for histologic review and immunohistochemistry. Given the budget limitation, we performed molecular analysis (EGFR mutations, ALK translocations, TS protein and mRNA expression) in 74 patients. Cases were collected from the p-Platform of the Foundation for Clinical and Applied Cancer Research (*FICMAC*, *Bogota*, *Colombia*) from May 2010 to January 2015. Our study was approved by Clinica del Country Institutional Scientific and Ethic Committee (R023-2014). All participants provided a written informed consent for inclusion and use of tumor tissue and clinical information. Written consents were stored in file.To secure confidentiality, a blinded independent staff member removed personal identification indicators from histologic material. The clinical data were compared and analyzed through codification.

### Microdissection, RNA isolation, and reverse transcription

One 10-μm-thick section was used for RNA extraction. The section was serial to three previous sections, each 4-μm thick, from the same formalin-fixed paraffin-embedded tumor block that was used for H&E staining to select appropriate neoplastic areas and for Ki_67_ and TTF1 immunohistochemistry. The 10-μm-thick section was dried at 56°C overnight, deparaffinized, and stained with Nuclear Fast Red solution (Nuclear fast red ab146372; ABCAM, San Francisco, CA, USA), and then it was hydrated through graded alcohols and microdissected.

### Quantitative real-time PCR

Quantitative PCR was performed using the ABI PRISM 7900HT Sequence Detection System (Applied Biosystems, Foster City, CA, USA) in a 384-well plate. All quantitative PCR mixtures contained 1 μL of cDNA template (<20 ng of reverse transcribed total RNA) diluted in 9 μL of distilled-sterile water, 1,200 nmol/L of each primer, 200 nmol/L of internal probe, and the TaqMan Gene Expression Master Mix (Applied Biosystems) for a final volume of 20 μL. The sequences of the primers and probes for TS and the *β-actin* reference gene were as follows: TS forward: 5′-GGCCTCGGTGTGCCTTT-3′, TS reverse: 5′-GATGTGCGCAATCATGTACGT-3′, TS probe: (FAM)-5′-AACATCGCCAGCTACGCCCTGC-3′-(TAMRA); E2F1 forward: 5′-CTCCAAGCCGTGGACTCTT-3′, E2F1 reverse: 5′-ACATCGATCGGGCCTTGT-3′, E2F1 probe: (FAM)-5′-CGGAGAACTTTCAGATCTCCCTTAAGAGCA-3′(TAMRA); *β-actin* forward: 5′-TGAGCGCGGCTACAGCTT-3′, *β-actin* reverse: 5′-TCCTTAATGTCACGCACGATTT-3′, *β-actin* probe: (FAM)-5′-ACCACCACGGCCGAGCGG-3′-(TAMRA). Cycling conditions were 50°C for 2 min, 95°C for 10 min followed by 46 cycles at 95°C for 15 s, and 60°C for 1 min. Baseline and threshold for cycle threshold (Ct) calculation were set manually using ABI Prism SDS 2.1 software. A mixture containing Human Total RNA (Stratagene, Santa Clara, CA, USA) was used as the control calibrator on each plate. EGFR mutations and ALK translocations were studied using COBAS 8100 (Cobas real-time PCR platform; Roche Diagnostics, Indianapolis, IN, USA) and fluorescence in situ hybridization (Vysis ALK Break Apart; Abbott Molecular, Des Plaines, IL, US). RT–PCR for mRNA TS was preformed for all study participants (n = 144); while immunohistochemistry was done for TS protein expression in 77 patients.

### Immunohistochemistry

Serial 5-μm-thick sections were collected into charged slides. After paraffin removal, rehydration through graded alcohols, and endogenous peroxidase activity quenching, the sections were treated in a pressure cooker for 5 min at 125°C, followed by a quick 10-s step at 90°C using EDTA buffer (pH 8.0). The slides were then incubated for 40 min at room temperature with primary mouse anti-TS antibody (clone 106; dilution, 1:100; Zymed), anti-Ki_67_ (clone MIB-1; dilution, 1:150; Dako) and TTF-1 (dilution, 1:75; anti-TTF1 antibody; ab76013, ABCAM, San Francisco, CA, USA). To assess TS immunostaining, the percentage of positive tumor cells was scored semiquantitatively as follows: low, if TS-positive cells were <10%; moderate, if TS-positive cells were between 11% and 39%; and high if TS-positive cells were >40%. The intensity of the expression index of Ki_67_ was divided into the following groups: <20%: low, 20–30%: high, >30%: very high.

### Statistical Analysis

For descriptive purposes, continuous variables were summarized as arithmetic means, medians and standard deviations. Categorical variables were reported as proportions with 95% confidence intervals (95% CIs). Inferential comparisons were performed using Student’s t test, χ^2^ or Fisher’s exact test were used to assess the significance among categorical variables. The time-to-event variables obtained from the Kaplan-Meier method were determined by log-rank tests. To test the association between TS mRNA expression (continuous variable) and clinicopathologic features (dichotomous variables), the Kruskal-Wallis and Mann-Whitney U tests were used. Statistical significance was determined as p≤0.05 using a two-sided test. All of the statistical analyses were performed using SPSS version 19.0 (SSPSS, Inc., Chicago, IL, US). In order to validate our results, we compared survival outcomes between our study population and a retrospective control group of 72 patients with advanced NSCLC who received CBP-PEM between 2011 and 2012. Groups were matched for demographic characteristics (S1 Table).

## Results

### Patients Characteristics

The cohort included 144 Colombian patients who received PCB followed by PB for maintenance therapy. The median age was 64 years (range, 32–86 years), 61% were female, and 65.3% had some history of tobacco exposure. All of the patients had predominantly adenocarcinoma on histologic analysis (97%). The performance status (PS) recorded by the Karnofski scale was >70% in 90% of the patients. Forty-three patients (30.5%) had brain metastases, 19 patients (13.5%) were treated with fractionated stereotactic radiosurgery (<6 lesions), 20 received cranial whole-brain radiotherapy, and 4 underwent both interventions. Only 3 patients had surgery. In these cases, bevacizumab was administered after the second or third cycle to minimize the risk of bleeding in every patient (Table 1).

**Table 1:** Patient characteristics.

Variable	N = 144 (%)
**Age**	
Mean, SD	64 (+/-10.5)
<40 years	2 (1.4)
41–60 years	57 (39.6)
61–80 years	75 (52.1)
>80 years	10 (6.9)
**Gender**	
Female	88 (61.1)
Male	56 (38.9)
**Histology**	
Adenocarcinoma	140 (97.2)
NOS	4 (2.8)
**Tobacco exposure**	
Smoker	15 (10.4)
Former smoker	79 (54.9)
Never smoker	39 (27.1)
No data	11 (7.6)
**Number of chemotherapy cycles**	
<4 cycles	61 (42.4)
>5 cycles	83 (57.6)
**EGFR mutation status**	
Mutated	9 (6.3)
Wild type	101 (70.1)
No data	34 (23.6)
**ALK traslocation**	
Present	3 (2.1)
Absent	70 (48.6)
Not available	71 (49.3)
**Number of organs affected**	
<2	125 (86.8)
>2	19 (13.2)
**Pleural metastases/Pleural effusion**	
Present	51 (35.4)
Absent	93 (64.6)
**Lung metastases**	
Present	83 (57.6)
Absent	61 (42.4)
**Brain metastases/Leptomeningeal disease**	
Present	43 (29.9)
Absent	101 (70.1)
**Bone metastases**	
Present	67 (46.5)
Absent	77 (53.5)
**Liver metastases**	
Yes	48 (33.3)
No	96 (66.7)
**Ki**_**67**_	
<20%	43 (29.9)
21–30%	44 (30.6)
>30%	57 (39.6)
**TTF1**	
Positive	98 (68.1)
Negative	30 (20.8)
ND	16 (11.1)
**TS protein expression levels (N = 74)**	
Negative	36 (48.6)
10–39%	18 (24.3)
>40%	20 (27.0)

Twelve cases were EGFR (n = 9) or ALK (n = 3) positive; these patients started the PCB regimen after genotyping confirmation and directed therapy availability. The mean number of PCB cycles before starting TK inhibitors was 2 and 8 for patients with EGFR mutations and ALK/EML4 translocations, respectively. Ten of these patients (83.3%) presented early complete or partial responses lasting 10.1 months (95% CI, 2.2–25.6) and had a median OS of 19.4 months (95% CI, 6.6–36.5). According to the Ki_67_ index, 43, 44 and 57 patients had an expression level of <20%, 20–30%, and >30%, respectively.

### Response and survival analysis

**Fig 1:**
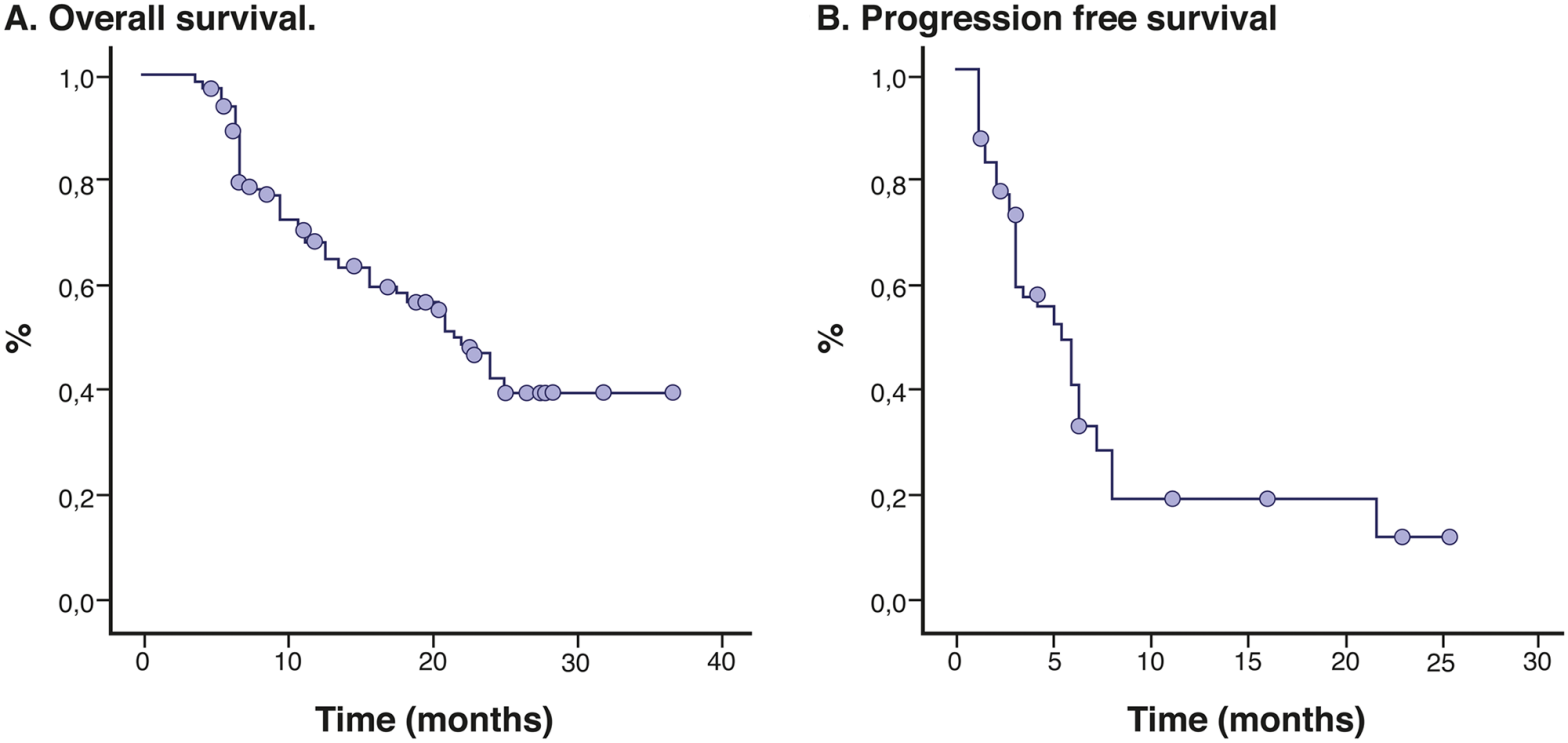
Kaplan Meier Curves for Overall Survival and Progression Free Survival.

**Fig 2:**
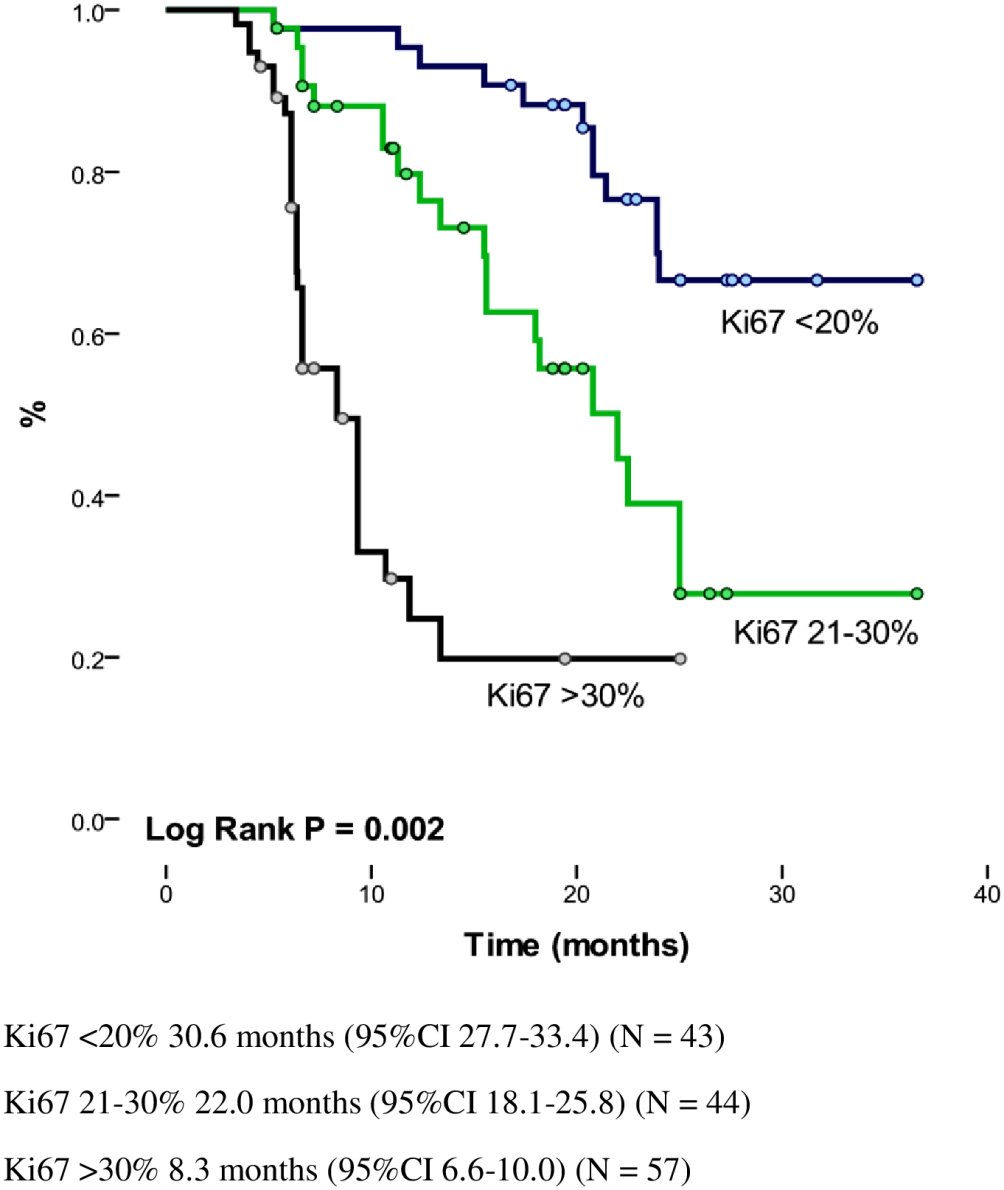
Overall survival according to Ki67.

The median follow-up was 13.8 months, and the median number of maintenance cycles was 6 (range, 1–32). Among the patients assessable for a response, the overall response rate (ORR) was 66% (95% CI, 47% to 79%) and 15.3% achieved stable disease. Clinical improvement and symptomatic relief were noted in 41% of the patients and clinical stability in another 40%. The response was greater in women (p = 0.002), among former and nonsmokers (p = 0.021), in those who had less than 10% weight loss within six months (p = 0.039), and in those who received > 5 cycles (p = 0.003). The median progression-free PFS and OS rates were 7.9 months (95% CI, 5.9–10.0 months) and 21.4 months (95% CI, 18.3 to 24.4 months), respectively (Fig 1). The median OS for the former and nonsmokers was slightly longer than for the smokers (22.2 vs. 16.4 months, respectively; p = 0.049). The prognostic significance of Ki_67_ was determined by a median OS of 30.6 months (95% CI, 27.7–33.4), 22.0 months (95% CI, 18.1–25.8) and 8.3 months (95% CI, 6.6–10.0) among those who had an expression level <20%, between 21 and 30%, and >30% (p = 0.002), respectively. The actuarial OS was longer among those who achieved a complete or partial response [median, 25.5 months (95% CI, 22.7–28.4)] versus the group of patients who progressed during PCB [median, 9.3 months (95% CI, 6.6–11.9); p = 0.0001]. PFS was longer among patients with better performance status Karnosfky Index (KI)>70%, median: 8.5 months (95% CI, 6.3–10.7) vs. KI<79%, median: 2.4 months (95% CI, 0.8–4.1); p = 0.0001], in those who received more than 5 cycles [median, 6.2 months (95% CI, 5.0–7.3) vs. ≤4 cycles 1.9 months (95% CI, 0.7–3.0); p = 0.0001], and in the group with the lowest expression level of Ki67 [<20%, median: 7.8 months (95% CI 0.0–18) vs. 20–30%, median: 6.0 months (95% CI, 5.2–6.7) vs. >30%, median: 2.9 months (95% CI, 1.8–4.1); p = 0.0001] (Fig 2).

In our study, we documented that Bevazizumab addition had a favorable impact insubjects who received PCB had an increase of 6.9 months in OS compared to the control group (CBP/Pem)(HR 0.64, CI95% 0.59–0.83; p = 0.016). The increase in OS was also observed (15 months) in patients with low expression of mRNA (HR 0.49, 95%CI 0.43–0.62; p = 0.002); similarly the OS in patients with low TS protein expression was 13.5 months (HR 0.76, 95%CI 0.69–0.88; p = 0.03). Regarding PFS, there was a 2.8 months difference between our study group vs. the historic control group, which was also significant (HR 0.82, 95%CI 0.80–0.94; p = 0.048). Likewise there was an increase in PFS in those who received PCB and had low TS protein expression compared to the control group (HR 0.97, 95%CI 0.94–1.32; p = 0.056).

### Toxicity

Grade 2 and 3 toxicities attributable to the PCB regimen included anemia (29% and 10%, respectively), fatigue (42% and 14%, respectively), febrile neutropenia (6%; grade 3 only), thrombocytopenia (26% and 3%, respectively), and thromboembolic disease (4.1% and 2.0%, respectively). The reported side effects (any grade) from bevacizumab included headache (20%), epistaxis (34%), hemoptysis (3.7%), muscular weakness (12%), transient erythema (19%), and hypertension (28%). Only one patient presented with bowel perforation and another had spontaneous tracheal rupture and secondary pneumomediastinum, likely secondary to bevacizumab.

### TS expression response and correlation with clinical outcomes

**Fig 3:**
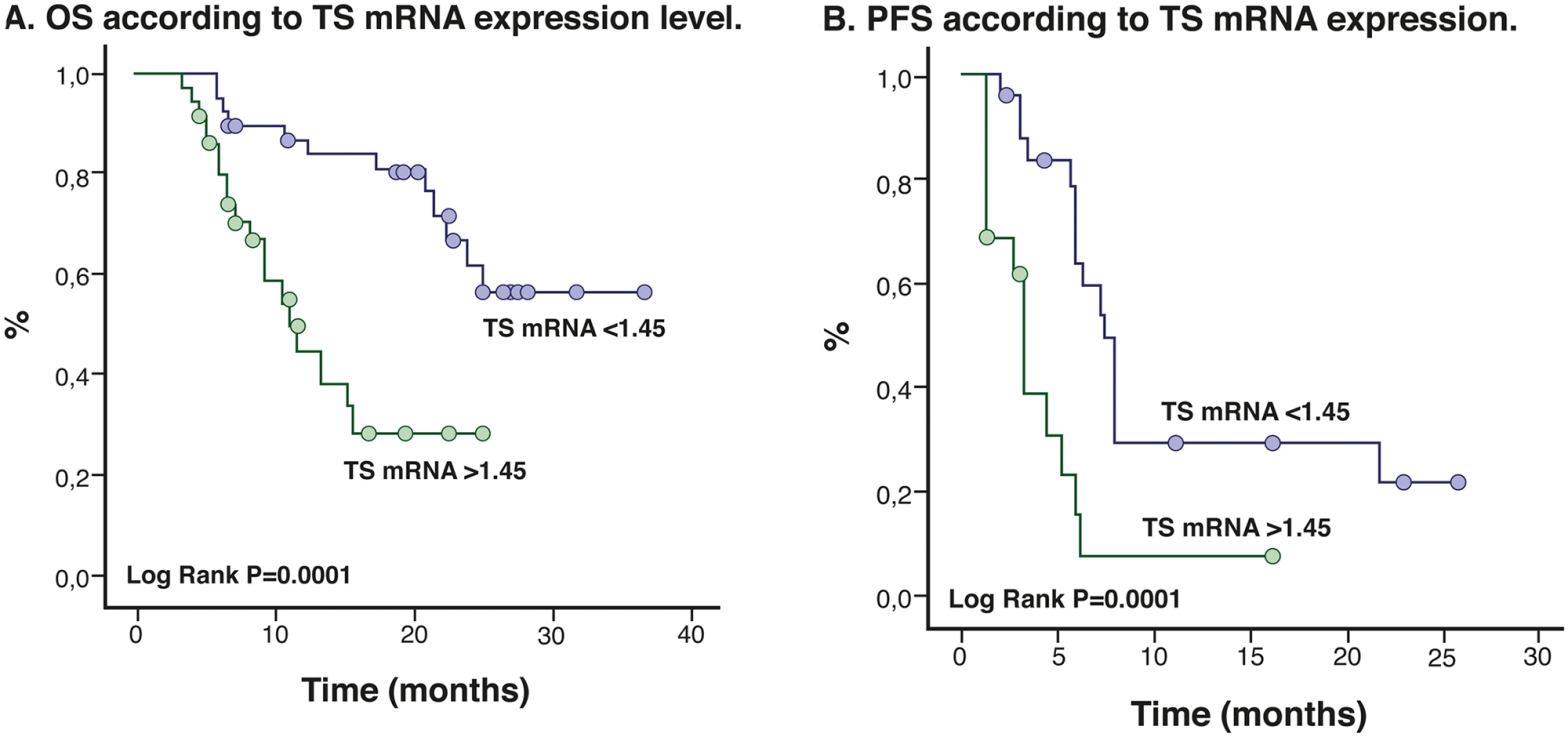
Kaplan-Meier Curves for Overall Survival and Progression Free Survival according to TS mRNA levels.

TS correlation was performed in 74 patients with a mean age of 64.1 years (range, 32–86 years), and 82.5% were former or nonsmokers and had a median TS mRNA level of 1.45 (range, 0.17–2.52). Stratifying the levels of TS mRNA by the median in 44.6% of the patients, the results showed high TS mRNA expression. The level of TS mRNA expression was not associated with tobacco exposure (p = 0.40), age (p = 0.37) or PS (p = 0.62). The TS mRNA levels were significantly higher among patients with Ki_67_ >30% (p = 0.0001) (Fig 2) and in those with a negative TTF1 (p = 0.006). The TS mRNA levels (p = 0.18) and protein expression (p = 0.51) did not influence the treatment response; however, OS was significantly higher in patients with the lowest TS mRNA levels [median, 29.6 months (95% CI, 26.2–32.9)] than in patients with higher levels [median, 9.3 months (95% CI, 6.6–12.0); p = 0.0001] (Fig 3a). The same trend occurred for PFS [lower TS mRNA levels vs. higher TS mRNA levels: median, 7.3 months (95% CI, 6.1–8.5) vs. 3.1 months (95% CI 2.6–3.5), respectively; p = 0.0001] (Fig 3b).

**Fig 4:**
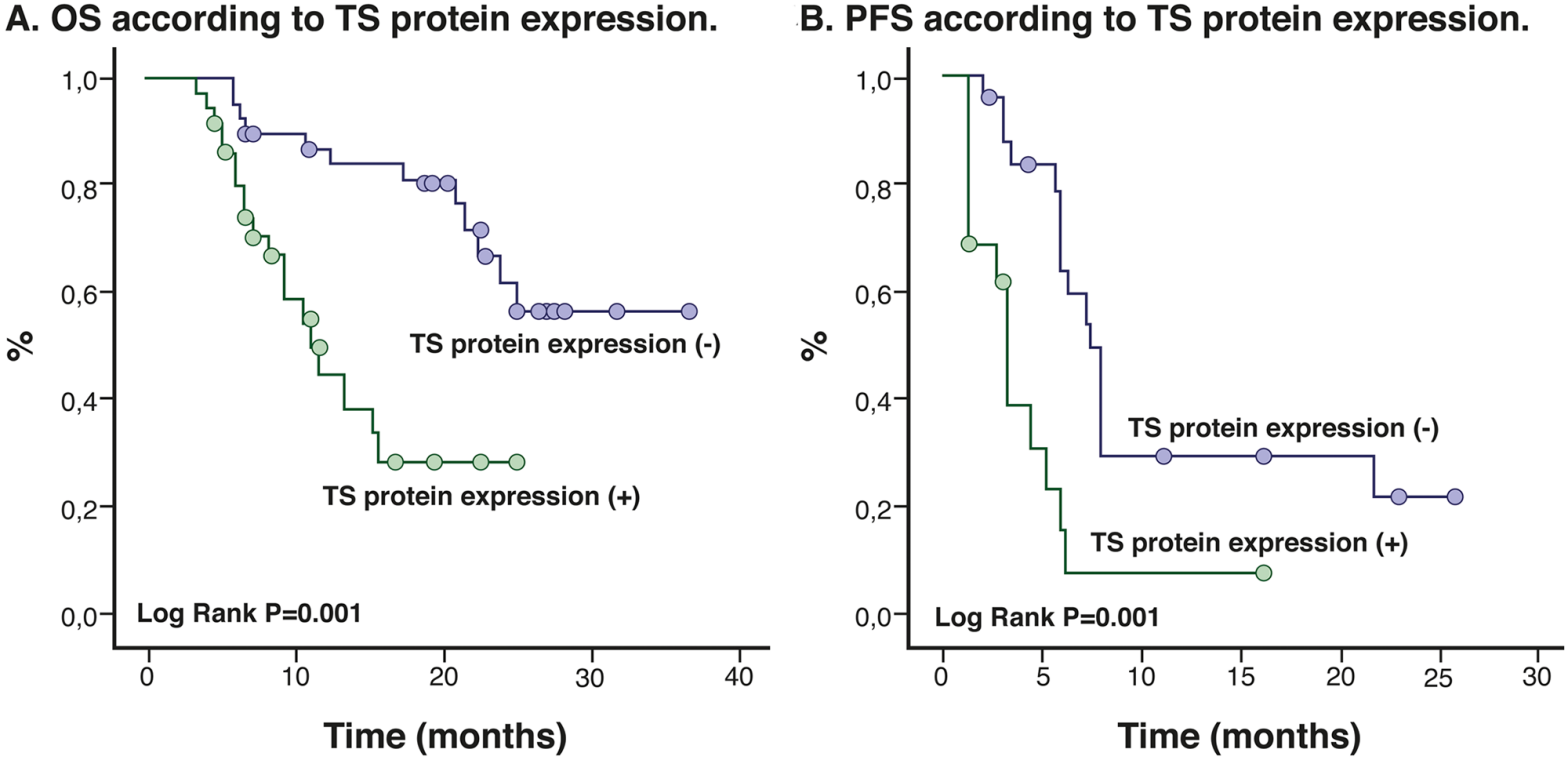
Kaplan Meier Curves according to TS protein expression.

**Fig 5:**
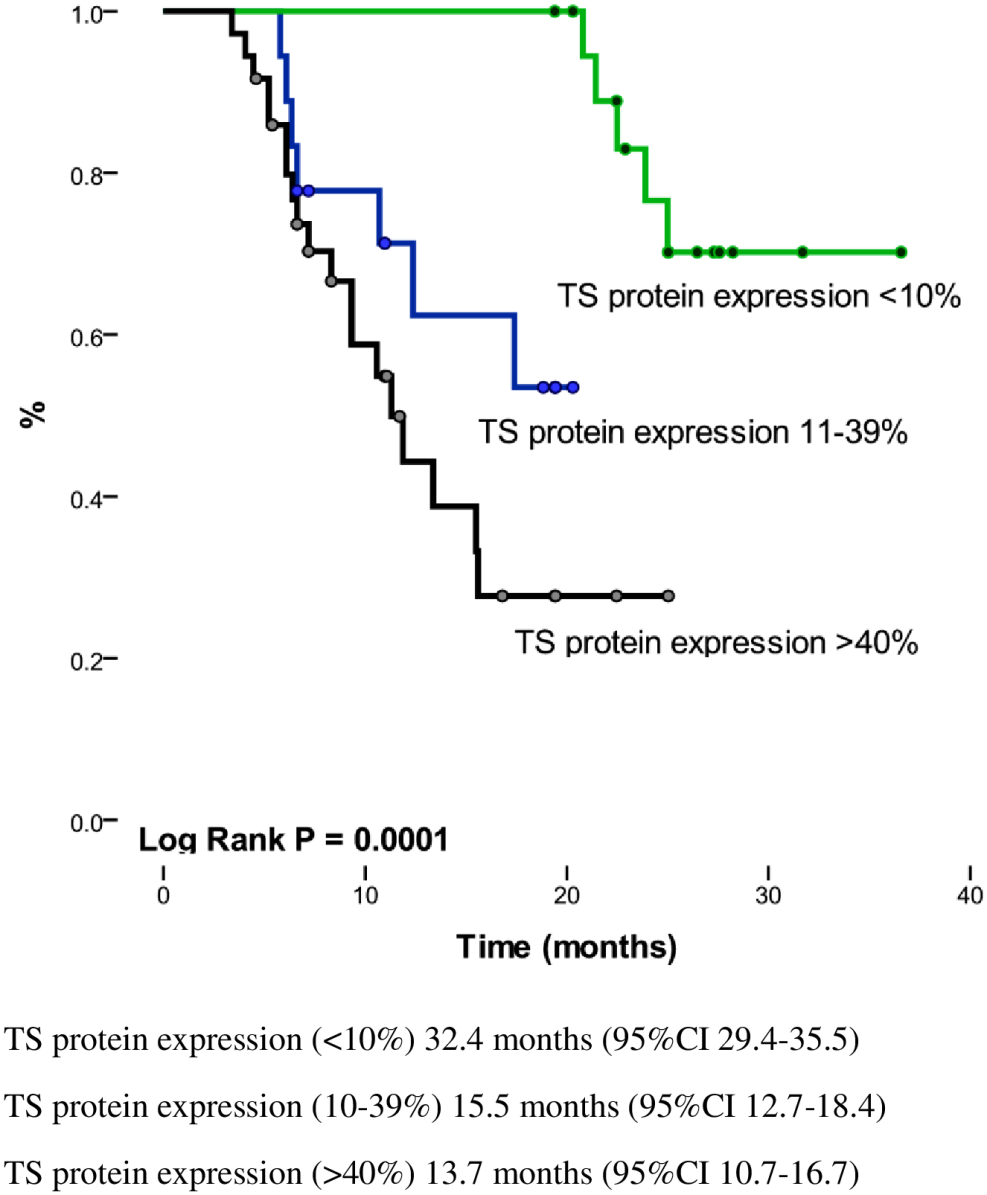
Overall survival according to TS protein expresson level (stratified).

In addition, after classifying patients according to TS expression levels (10%, 11% to 39%, and > 40%), we found that OS and PFS decreased with increasing TS expression levels (log-rank p = 0.0001; Fig 4a and 4b). The OS when TS expression is ≤10% was 28 months (95%CI 24.1–31.8), for the subgroup with TS expression level between 11% to 39% OS was 15.5 months (95% CI, 12.7–18.4); p = 0.0001], while the median OS for the TS >40% subgroup decreased significantly to 13.7 months (95% CI, 10.7–16.7) (Fig 5).

Importantly, in multivariate analysis, the TS mRNA, TS protein expression level and number of maintenance cycles modified OS (p = 0.017, p = 0.047 and p = 0.002, respectively). Moreover, we found a significant correlation between the TS mRNA levels and protein expression (p = 0.002).

## Discussion

To our knowledge, this is the first study describing the efficacy and safety of PCB chemotherapy in a Hispanic population with non-squamous NSCLC. The median OS (21.4 months) in our series was superior to that of previous reports with doublet platinum-based plus bevacizumab regimens with maintenance (median OS, 11.7 to 17.1 months) [24,25,31]. Barlesi *et al*., in a phase III trial, demonstrated that induction chemotherapy with cisplatin/pemetrexed/bevacizumab followed by maintenance with PB had superior PFS rates (difference of 3.6 months) compared with maintenance therapy with bevacizumab alone in patients with non-squamous NSCLC. The authors documented that patients who received maintenance therapy with PB and had stable disease during induction regimen had a tendency to have better OS (≈4 months); however, this improvement, was not statistically significant [24, 32]. Furthermore, the authors reported that PFS in the PB arm was 7.4 months, which is slightly inferior to our results (7.9 months). After analyzing other endpoints, Barlesi *et al*. found an ORR of 55% (95% CI, 46–59.1%), also inferior to our data (ORR of 65% with 95% CI, 47%-79%) [32]. In contrast, in our study there were no differences in the proportion of patients who received second-line therapy (treatment after progression; 70.5% vs. 86%), which would not explain the difference in survival between these two studies [32].

Similarly, Patel *et al*. compared PCB as an induction regimen followed by PB (study arm) with carboplatin/paclitaxel/bevacizumab as induction chemotherapy followed by maintenance with bevacizumab (control arm). The authors did not find significant differences in the primary endpoint (OS); nevertheless, they described a 0.4-month PFS improvement favoring the study arm [25]. After comparing the endpoints between Patel’s study, we documented superior OS and PFS (12.6 vs. 21.4 months and 6 vs. 7.9 months, respectively), additionally we found better ORR (34.1 vs. 65%). In terms of PFS and ORR, it is likely that our improved outcomes are the result of the extended OS. However, since we did not have a control arm, comparing our data with other studies has important inherent limitations.

In a similar study design, Liu and Wu retrospectively reviewed the effect of cisplatin, pemetrexed, and bevacizumab with a median of 4 cycles. The investigators did not administer maintenance therapy and observed an equal frequency of partial response and stable disease. The ORR was 43%, the median PFS was 5.2 months (95% CI, 3.7 to 6.7 months), and the median OS was 11.4 months (95% CI, 8.8 to 13.9 months) [38]. In contrast, we only administered carboplatin, which is the standard of care, and compared with cisplatin, has a better safety profile [33].

PRONOUNCE is a phase III trial with a similar methodologic design to that of the Patel study. In this trial, 361 patients were randomized to receive carboplatin/pemetrexed followed by pemetrexed as maintenance therapy or PCB followed by bevacizumab as maintenance therapy [34]. The primary endpoint of this study was PFS without grade 4 toxicity (PFS G4); the authors did not find differences in the primary or secondary endpoints (OS, PFS) between the two treatment strategies. Compared with the PRONOUNCE trial, we documented better clinical outcomes, however our study lacked a control arm.

The toxicity profile encountered in our cohort is similar to that of previous studies. According to the literature, PCB followed by PB is a well-tolerated regimen with acceptable toxicity that is rarely fatal. In our study, grade 3 toxicity was the most commonly documented hematologic toxicity. We commonly reported neutropenia and anemia, the frequencies of which were similar to those found in other studies [23,25,32,34]. In accordance with other trials, the most frequent non-hematological grade 3 toxicity was fatigue (11% vs. 2.4 to 10.9%)[25,32,34]. Neither hemorrhagic nor thromboembolic grade ≥3 events associated with bevacizumab were present in our series; however, we observed a case of gut perforation and a case of tracheal rupture.

TS expression is recognized as a predictive factor for the response to pemetrexed. Two meta-analyses have evaluated such relationship; Wang *et al*. analyzed 526 NSCLC patients from eight clinical trials that assessed TS expression and the ORR to pemetrexed. The authors documented superior ORR in patients with low TS expression (Odds Ratio = 0.45; 95% CI, 0.29–0.70; p = 0.0004). A low level of TS expression was also associated, with better PFS and OS, albeit the latter was not statistically significant [35]. Similarly, Liu *et al*. evaluated the associations between TS expression, PFS and OS in eight studies of patients treated with pemetrexed. The authors also suggested that patients with low TS expression had better PFS and OS than patients with higher TS levels (PFS: HR = 0.63; 95% CI, 0.52–0.76; OS: HR = 0.74, 95% CI, 0.63–0.88) [36]. We also reported that low TS expression was associated with better clinical outcomes (OS) and confirmed the usefulness of TS expression as a prognostic factor. However in the population we analyzed (n = 79), TS expression (mRNA levels and protein expression) did not influence the treatment response. Likewise, a recently published Phase II trial by Sun *et al*. evaluated TS as a predictive marker for the clinical outcome of NSCLC patients after treatment determined the status of TS expression. A total of 341 subjects were stratified according to TS expression (defined as negative or positive) and received either pemetrexed/cisplatin or gemcitabine/cisplatin for a maximum of 6 cycles until disease progression. The authors documented that pemetrexed/cisplatin was superior to gemcitabine/cisplatin based on the response rate and PFS, (47% vs. 21%, P = 0.008) in the TS-negative group. Moreover, the median OS time of all TS-negative group patients was significantly longer than the OS time of patients in the TS-positive group (30.3 vs. 15.2 months, respectively; HR, 0.50; 95% CI, 0.36 to 0.70) [37]. Consistent with our cohort results, this finding suggested that low TS expression is associated with a better OS.

Additionally, our study also supported Ki_67,_ as a prognostic factor; since lower Ki _67_ expression was associated with longer OS. This is consistent with a previous meta-analysis of 32 studies involving 5600 Asian patients; in such study the authors established that elevated Ki _67_ was also linked to poorer outcomes in NSCLC [38].

Our study limitations include lack of a control arm and small sample size; in a forthcoming study it will be important to analyze the association of tumor heterogeneity and the predictive value of both TS and Ki 67.

## Conclusions

PCB followed by PB was effective and safe in our study group. This regimen was associated with acceptable toxicity and with prolonged OS. In our cohort both Ki_67_ and TS expression were important prognostic biomarkers.

## Supporting Information

S1 Dataset Patient data base. (SAV)

S1 Table Characteristics of control group. (DOCX)

## Abbreviations

PCB: Pemetrexed, carboplatin and bevacizumab
PB: Pemetrexed and Bevacizumab
TS: Thymidylate synthase
NSCLC: Non-Small Cell Lung Cancer
ORR: Overall response rate
PFS: Progression-free survival
OS: Overall survival
LC: Lung cancer
IARC: International Agency for Research on Cancer
